# A case report of targeted therapy with apatinib in a patient with advanced gastric cancer and high serum level of alpha-fetoprotein

**DOI:** 10.1097/MD.0000000000004610

**Published:** 2016-09-16

**Authors:** Xue-Ru Zhu, Mei-Ling Zhu, Qing Wang, Wen-Ji Xue, Yi-Wei Wang, Rui-Fen Wang, Si-Yu Chen, Lei-Zhen Zheng

**Affiliations:** a Department of Oncology; b Department of Pathology, Xin Hua Hospital affiliated To Shanghai Jiaotong University School of Medicine, Shanghai, China.

**Keywords:** apatinib, gastric cancer, serum AFP, targeted therapy

## Abstract

**Background:**

Alpha-fetoprotein (AFP) is an important marker for hepatocellular carcinoma, and the detection of serum AFP is currently the principle method for the diagnosis of hepatocellular carcinoma. The prevalence of gastric cancer (GC) with high level of serum AFP is extremely rare, but has unique clinical features.

**Case summary:**

We herein present a rare case with GC and high level of serum AFP. A 64-year-old Chinese female underwent gastrectomy was diagnosed as gastric adenocarcinoma and the pathological stage was T_1b_N_0_M_0_, IA. With the progression of disease, the tumor widely metastasized and the serum AFP level increased progressively with the highest level of 3396 ng/mL. She successively entered into 3 lines palliative systematic chemotherapy and fourth-line targeted therapy of apatinib, a small molecule tyrosine kinase inhibitor targeting vascular endothelial growth factor receptor-2. Although previous studies suggested that the prognosis of this special type of GC was poor, this patient lived for 22 months after tumor transfer. Apatinib kept her progression-free survival for 5 months, and the overall survival was 4.5 years.

**Conclusion:**

So, we speculate that maybe we can focus apatinib on serum AFP elevated GC patients.

## 1. Introduction

Serum alpha-fetoprotein (AFP) level initially elevates due to the product of fetal liver, yolk sac and some fetal gastrointestinal cells and decreases rapidly after birth.^[1]^ In clinical practice, we usually consider AFP as a useful tumor marker for monitoring the patients of hepatocellular carcinoma (HCC) or yolk sac tumor.^[2]^ Previous studies showed that the elevation of AFP might also be found in patients with tumors of other organs and abnormal serum AFP elevation in gastric cancer is the most common among all extrahepatic tumors its percentage ranges between about 1.3% and 15%.^[3]^ Although the influence of serum AFP on gastric cancer remains unclear,^[4]^ some retrospective studies have revealed its tendency to metastasize to multiple lymph nodes, and the prognosis was poor.^[5,6]^

Apatinib is a small molecule tyrosine kinase inhibitor targeting vascular endothelial growth factor receptor-2 (VEGFR-2), which functions as antiangiogenesis and has been recommended as a third-line treatment for metastatic gastric cancer patients.^[7]^ So far, there is no literature reported using it for gastric cancer with high level of serum AFP. We herein report a unique case of apatinib treating gastric cancer with serum AFP significant increasing.

## 2. Case report

**Figure 1:**
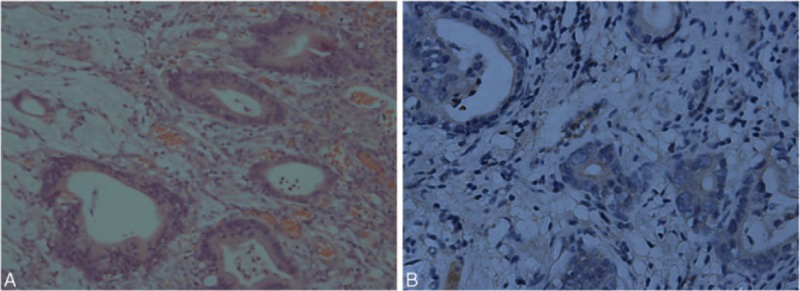
(A) Hematoxylin and eosin staining, magnification, ×200: the major histological type is adenocarcinoma. (B) Immunohistochemical staining for α-fetoprotein (negative) in the adenocarcinoma. Magnification, ×400.

**Figure 2:**
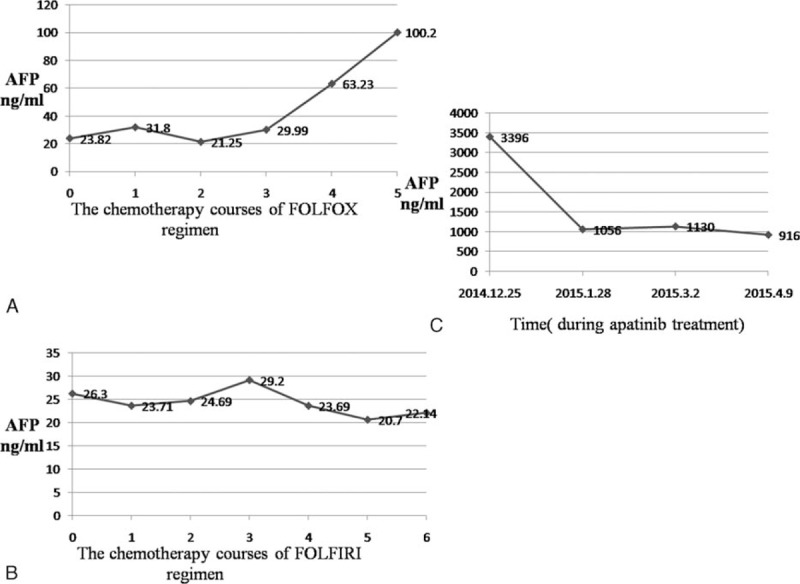
The change of serum AFP level. (A) (Chemotherapy regimen with FOLFOX): the levels of AFP in serum were gradually increased; (B) (chemotherapy regimen with FOLFIRI): the levels of AFP in serum were stable; (C) (targeted therapy with apatinib): the levels of AFP in serum decreased sharply. AFP = alpha-fetoprotein.

**Figure 3:**
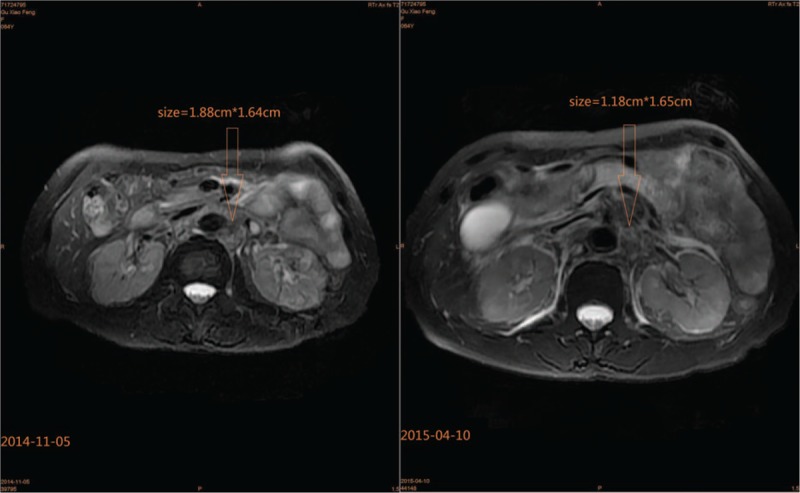
MRI examination of abdomen, showing the mass of retroperitoneal lymph node. Before the targeted therapy of apatinib, the size was 1.88 × 1.64 cm, after 4 months therapy, it reduced to 1.18 × 1.65 cm. MRI = magnetic resonance imaging.

A 64-year-old woman suffered with repetitive upper abdominal dull pain for almost 3 months. Gastroscopy examination suggested she might get gastric cancer. On December 17, 2010, Billroth I gastrectomy was performed, and the pathological diagnosis was gastral tubular adenocarcinoma (Fig. 1A), and the stage wasT_1b_N_0_M_0_, stage IA. The patient received no therapy until August 12, 2013, the following detection revealed that the patient's serum AFP level elevated from normal range to 23.83 ng/mL (normal value range is 0–7 ng/mL), but the immunohistochemical staining for α-fetoprotein in the adenocarcinoma was negative (Fig. 1B). The liver function was normal and negative results denied hepatitis. abdominal computed tomography examination showed anastomotic recurrence and retroperitoneal lymph node metastasis. Then, we gave her 3 lines intravenous chemotherapy with FOLFOX, FOLFIRI, and TP regimens, and obtained partial response for 1.5 months, stable disease for 5 months and progressive disease, respectively. Then, the target therapy-apatinib (850 mg po qd) was given and the patient's serum AFP level decreased the most among all of these therapies from 3396 to 916 ng/mL (Fig. 2) and the videographic examine showed that disease was stable (Fig. 3). On April 10, 2015, because the side effects of diarrhea, proteinuria, and blood pressure increasing, the dose decreased to 750 mg po qd. Unfortunately, on May 18, 2015, severe jaundice occurred and we had to stop apatinib. The patient finally died of terminal gastric cancer on June 12, 2015, the overall survival (OS) was 4.5 years and survival time was 22 months from tumors occurred distant metastasis.

## 3. Discussion

Before our case discussion, we should explicate 2 conceptions, they are AFP-producing gastric cancer and hepatoid adenocarcinoma. In 1970, Bourreille et al^[8]^ first reported a case of AFP-producing gastric cancer, which refers to a type of gastric cancer that AFP is positive in the immunohistochemical staining of pathological specimen. In 1985, Ishikura et al^[9]^ first described hepatoid adenocarcinoma, which showed a histologic appearance typical of HCC, including solid, trabecular, and pseudogranular structure; tumor cells were round or polygonal in shape.^[10]^ Different with above 2 kinds of gastric cancer, our patient's feature was just with serum AFP elevation.

In our case, the patient suffered from an early stage gastric cancer and serum AFP level was normal initially. Thirty-two months after gastrectomy, tumor occurred metastasis and serum AFP level increased. Three lines systematic chemotherapies were done, and the longest stable time was 5 months. To our surprise, the fourth-line targeted therapy with apatinib still got 5 months progression-free survival and serum AFP levels decreased sharply, which was the biggest fall during overall healing process.

Recent years, although considerable improvement in medicine science, the prognosis of advanced gastric cancer is poor and average survive is <1 year.^[11]^ In previous studies, first-line chemotherapy can provide a 6 months survival benefit for patients with advanced gastric cancer, second-line chemotherapy with irinotecan or docetaxel added only about 1.5 months to the OS compared with best supportive treatments.^[12–15]^ And some studies have revealed that serum AFP elevation gastric carcinoma has poorer prognosis than common gastric cancer.^[16]^ Compared with aforementioned data, the patient OS was obviously longer than most literature reports and fourth-line targeted therapy with apatinib also provided 5 months progression-free survival benefit for the patient.

Apatinib is a small molecule tyrosine kinase inhibitor targeting VEGFR-2, its function is anti-angiogenesis. In a phase III study, apatinib showed a good efficacy for chemotherapy-refractory advanced gastric cancer patients.^[7]^ However, there were no studies using apatinib in serum AFP elevation gastric cancer. Kamei et al^[17]^ did a contrast study to compare the vascular endothelial growth factor-C (VEGF-C) expression frequency between AFP positive and negative gastric cancer groups, they found VEGF-C expression frequency significantly higher in AFP positive group than negative group. So they thought that AFP may upregulate expression of VEGF-C by interacting with transcription factors or key protein. VEGF-C is one of the VEGF isoforms, it serves as a lymphangiogenic factor and generates excess lymph vessels.^[18]^ VEGFR-2 is one of the receptor of VEGF-C and it expresses on vascular epithelial and lymphoepithelial. Recently, Yonemura et al^[19]^ found that the VEGF-C expression correlated strongly with poor prognosis of gastric cancer. From the aforementioned, we deduce that the invasiveness and poor prognosis of serum AFP elevation gastric cancer may be correlated with AFP upregulating the expression of VEGF-C. And by inhibiting VEGFR-2, apatinib can decrease the expression of AFP and then improve the prognosis. Besides the explanation of mechanisms, relative clinical practice also had good news. SHARP, a phase III trial, reported that sorafenib improved the OS of HCC patients 2.8 months than placebo,^[20]^ which was the first agent that improved the OS of patients with advanced HCC. Sorafenib is a multikinase inhibitor, similarly to apatinib, it also acts on VEGFR-2. However, another large and latest experiment, STORM, which used sorafenib as adjuvant chemotherapy for HCC, showed that with regard to RFS, OS or time to recurrence, there is no significant treatment effect with sorafenib.^[21]^ These contradictory outcomes may due to the difference of individuals, ethnic and stages of HCC between the 2 trials. So, maybe, we can focus apatinib on gastric cancer patients with high serum AFP level and make further research.

Yet, if we focus apatinib on serum AFP elevated gastric cancer patients, there are still some questions needed to be considered: First, when is the best time to use apatinib for patients with serum AFP elevation gastric cancer? Secondly, which is the optimal value? If we use apatinib based on the level of serum AFP. Thirdly, whether apatinib can be used as the second-line treatment for patients with serum AFP elevated gastric cancer? The last but most important, further clinical trials and experiments are needed to prove our deduction and answer all of the questions.

## 4. Conclusion

Serum AFP elevated gastric cancer is a rare type of gastric cancer with doughty invasiveness and poor prognosis. In most literatures, the gastric cancer patients with serum AFP elevation were given various of chemotherapy regimens but the outcome were not good enough; however, few reports were about the efficacy of apatinib. According to the mechanisms of AFP upregulates the expression of VEGF-C, the inhibitors of VEGFR or VEGF might be potential drugs to cure this special type of gastric cancer patients. And although this type of disease is rare, it deserves further studies.
